# Cardiovascular Risk Factors Have Larger Impact on Endothelial Function in Self-Reported Healthy Women than Men in the HUNT3 Fitness Study

**DOI:** 10.1371/journal.pone.0101371

**Published:** 2014-07-03

**Authors:** Eli-Anne Skaug, Erik Madssen, Stian Thoresen Aspenes, Ulrik Wisløff, Øyvind Ellingsen

**Affiliations:** 1 Department of Circulation and Medical Imaging and KG Jebsen Center for Exercise in Medicine Norwegian University of Science and Technology, Trondheim, Norway; 2 Department of Cardiology, St Olavs Hospital, Trondheim, Norway; Stellenbosch University, South Africa

## Abstract

**Background:**

Several studies suggest that cardiovascular risk factors comprising the metabolic syndrome have larger effects on the development of cardiovascular disease in women than in men. A recent study in self-reported healthy subjects demonstrated a marked gender difference in endothelial dysfunction that may be an important precursor of manifest cardiovascular disease. The aim of the present study was to determine whether the association between endothelial function and cardiovascular risk factors is different in self-reported healthy women compared to self-reported healthy men.

**Methods and Results:**

Associations between endothelial function (flow mediated dilation, FMD, of the brachial artery measured by ultrasound), anthropometric variables, peak oxygen uptake (VO_2peak_), blood pressure, serum lipids, blood glucose and a questionnaire on general health and lifestyle including smoking status were studied by logistic and linear regression in 2 528 women and 2 211 men aged 20–89 years, free from self-reported cardiovascular disease. In women with hyperglycemia, endothelial dysfunction (FMD ≤0%) occurred twice as frequently as in male counterparts. The presence of the metabolic syndrome, high blood pressure and low VO_2peak_ increased the prevalence of endothelial dysfunction more in women than in men.

**Conclusion:**

Endothelial dysfunction is more strongly associated with cardiovascular risk factors in self-reported healthy women than in self-reported healthy men. This finding could explain why the metabolic syndrome, and especially hyperglycemia, is associated with higher cardiovascular risk and a worse prognosis in women.

## Introduction

Endothelial dysfunction (ED) predicts vascular events in subjects with and without established cardiovascular disease (CVD) [1], [2], and is associated with risk factors for atherosclerosis [3], [4], [5], [6]. A recent survey of self-reported healthy respondents from the HUNT3 Fitness study [7] demonstrated that women have better endothelial function than men in all age groups up to 65–70 years of age. This gender difference corresponds to the age-related incidence of CVD.

The metabolic syndrome increases the risk of cardiovascular disease and worsens the prognosis more in women than in men [8], [9], [10], [11], [12]. Furthermore, ED seems to potentiate the negative effect of the metabolic syndrome on mortality and cardiovascular events in women [13], [14]. These observations are consistent with the notion that endothelial function may be a pivotal factor explaining gender differences in CVD. The aim of the present study was to determine whether the association between endothelial function and cardiovascular risk factors is different in self-reported healthy women compared to self-reported healthy men.

## Study Population and Methods

### Inclusion and exclusion

Associations between endothelial function and cardiovascular risk factors were studied in a subset of the HUNT3 health survey. HUNT3 covered 52% of the population in Nord-Trøndelag County, which is considered a close average of the general Norwegian population. The selection and characteristics of 4 739 adult respondents from the HUNT3 Fitness study and their age and gender distribution of flow mediated dilation (FMD) was recently reported [7]. Briefly, the respondents completed health-related questionnaires including physical activity habits, and underwent measurements of endothelial function (flow mediated dilation; FMD), peak oxygen uptake (VO_2peak_), and a brief clinical examination (7). Inclusion criteria were participation in the HUNT3 main study and informed consent to participate in the HUNT3 Fitness Study. Exclusion criteria were self-reported episodes of obstructive breathing or dyspnea for the past 12 months, present or former asthma, chronic obstructive pulmonary disease, sarcoidosis, cancer, and established CVD, including cerebral and peripheral arterial disease, angina pectoris and previous myocardial infarction, contraindications towards physical activity, and use of antihypertensive or vasoactive medication known to influence endothelial function. Respondents with cardiac arrhythmias during testing were excluded.

Of 30 165 participants who were “self-reported healthy” according to these criteria, 18 608 were randomly invited to the Fitness Study [7]. Out of the 5 636 that were included at attendance, 4739 (2 528 women and 2 211 men) were willing to participate and completed endothelial function testing, comprising our study population. A flowchart of inclusion, and characteristics of selection and the study population, including risk profile, are presented in our previous publication [7].

### Approvals

The Regional Ethics Committee, the Norwegian Data Inspectorate, and the Ministry of Health and Care Services approved of the HUNT3 Fitness study. The Study Protocol conformed to the Helsinki declaration, and written informed consent was obtained from all participants.

### Endothelial function test

FMD was assessed in the left brachial artery by a 3-point echocardiography system (Vivid i, GE Healthcare, USA) with a high-resolution 12 MHz array transducer. A blood pressure cuff was placed on the forearm [15], [16] and the participants were in supine position in a quiet and dark room. Blood flow was estimated by pulsed Doppler velocity signals. The brachial artery was imaged at baseline after ten minutes of supine rest, and 60 seconds after cuff deflation following 5 minutes of arterial occlusion at 250 mm Hg. Arterial diameter was measured from intima to intima at the peak of the R-wave to avoid confounding of cyclic changes. The mean of three consecutive diameter measurements was recorded.

FMD was defined as percent change in vessel diameter, calculated as (post occlusion diameter minus baseline diameter) divided by baseline diameter. ED was defined as FMD ≤0%. Shear rate yielded the same patterns for age and gender (7), therefore only unadjusted values were included in the present analyses. Interobserver analysis of recordings gave a mean difference ranging from −1.236 (95% CI: −5.377 to 2.904) to 2.250 (95% CI: −1.348 to 5.848) using the Bland–Altman plot, with Pitman’s Test of difference in variance ranging from r = 0.008 (n = 81, p = 0.942) to r = −0.846 (n = 82, p = 0.000).

All subjects received written information in advance and were asked to fast and refrain from coffee and tobacco the last four hours before test, but this was not achieved in all respondents since recordings were taken throughout the entire day. Still, there was no difference in FMD between fasting and non-fasting subjects. As detailed below, the criterion for hyperglycaemia was adjusted to account for suboptimal fasting conditions.

### Clinical measures

Resting heart rate was recorded as the lowest heart rate during supine rest for 10 minutes. Blood pressure was measured (Critikon Dinamap 845XT, GE Medical Systems, USA) according to guidelines [17]. Height and weight were measured to the nearest centimeter (cm) and kilogram on a combined scale (Model DS-102, Arctic Heating AS, Norway), and body mass index (BMI) was calculated. Waist circumference was measured to the nearest cm at the height of the umbilicus, and limits between normal waistline and overweight set according to the definition for the metabolic syndrome [18], as referred below. For BMI, overweight was defined as BMI 25 kg/m^2^–30 kg/m^2^ and obesity as BMI>30 kg/m^2^. All blood samples were analyzed by photometric methods (Architect ci8200, Abbott Laboratories, IL, USA). VO_2peak_ was measured by ergospirometry during treadmill running [19] and physical activity index calculated as described by Aspenes et al [20].

### CVD risk factors

The following cardiovascular risk factors were assessed: Systolic and diastolic blood pressure, waist circumference, body mass index, total cholesterol, high-density lipoprotein (HDL) cholesterol, blood glucose, serum triglycerides, fitness (VO_2peak_), physical activity index [20], smoking (yes or no), and the cluster of risk factors comprising the metabolic syndrome. LDL cholesterol was not measured in HUNT3 health survey. The risk profile was calculated according to ESC Score for population at low cardiovascular risk based on the ratio between total and HDL cholesterol [21].

The metabolic syndrome was defined according to current recommendations [18], as the presence of at least three of the following; elevated waist circumference (≥94 cm in men, ≥80 cm in women), serum triglycerides ≥1.7 mmol/L, low HDL-cholesterol (<1.0 mmol/L in men, <1.3 mmol/L in women), elevated blood pressure (systolic blood pressure ≥130 and/or diastolic blood pressure ≥85 mm Hg) and elevated fasting glucose. To account for blood sampling less than four hours of fasting in the majority of participants, the glucose criterion was changed from ≥5.6 mmol/L, to ≥7.8 mmol/L, corresponding to the impaired glucose tolerance criterion [22].

### Statistical analysis

Since our previously published analyses of the Fitness Study cohort demonstrated consistent age and gender differences in FMD [7], data from women and men were analyzed separately, and regression analysis repeated with and without adjustment for age. Means with 95% confidence intervals were calculated for baseline brachial artery diameter and FMD. Baseline characteristics were compared between groups using a 2-tailed independent t-test for continuous variables. Fisher's z-transform method was used to compare correlation coefficients between the genders. Associations between FMD, ED and risk factors were analyzed by logistic regression and multiple linear regression. Trends across ordered groups were identified by test for linearity, by nonparametric equality-of-medians test, and by the Wilcoxon rank-sum test corrected for ties. Results are reported from nonparametric analyses unless else is specified. Cases with missing values were excluded from multivariate analyses. Regression analyses were adjusted for smoking status. SPSS software version 17 was used for all statistical analyses.

## Results

### Metabolic syndrome and FMD

**Figure 1:**
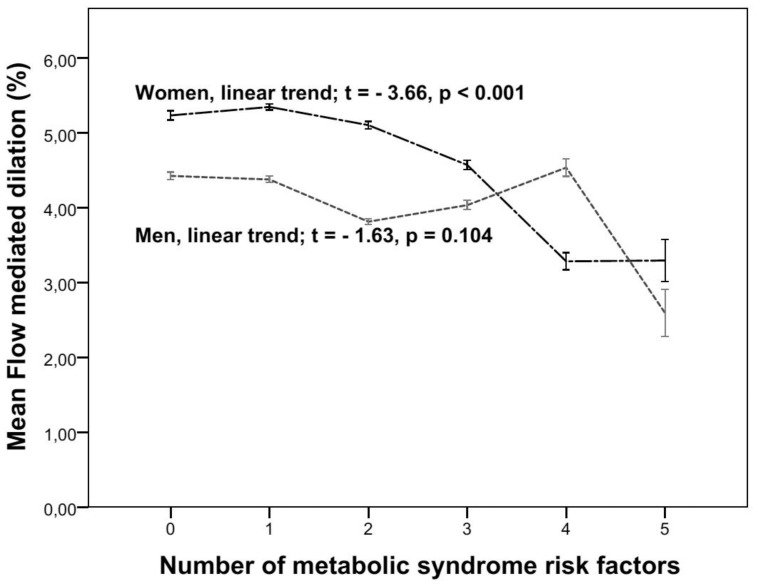
Flow-mediated dilation with number of risk factors present. Mean flow mediated dilation adjusted for age and its dependence on risk factors included in the definition of the metabolic syndrome (waist obesity, hypertension, hyperglycemia, low HDL, and high trigycerides) are shown separately for women and men. Vertical lines indicate 95% confidence intervals.

In women, FMD was lower (p<0.001) and the prevalence of ED was higher (p = 0.013) with the presence of the metabolic syndrome (Table 1, 2). There was also a positive association for the prevalence of ED (p = 0.009), and a linear negative association for FMD (p<0.001) respectively, with the number of metabolic syndrome components present (Fig. 1). In men, there was only a trend for association between FMD and the number of metabolic syndrome components (p = 0.10). In women with the metabolic syndrome, its presence could explain 25% of the variance of FMD (effect of 3 versus 0 risk factors; r = 0.52, p<0.001).

**Table 1:** Flow-mediated dilation and endothelial dysfunction by cardiovascular risk factors stratified by gender and metabolic syndrome.

	WOMEN					MEN				
	n	Age,(CI)	FMD,(CI)	FMD_adj_(CI)	ED,%	n	Age,(CI)	FMD,(CI)	FMD_adj_(CI)	ED,%
**All subjects**										
No MetSyn	2181	47.6	5.5^a^	5.2	15.4	1776	48.3	4.3^b^	4.2	17.9
		(47.5–48.8)	(5.27–5.65)	(5.21–5.27)			(47.5–48.8)	(4.12–4.47)	(4.15–4.21)	
MetSyn	347	55.0	4.5^c^	4.3	19.9	435	52.6	4.3^d^	4.1	17.5
		(53.6–56.4)	(4.05–4.84)	(4.24–4.35)			(51.5–53.8)	(3.90–4.65)	(4.07–4.18)	
**Elevated waist circumference**										
No MetSyn	1461	48.9	5.5^e^	5.3	15.3	786	51.7	4.2^f^	4.1	17.1
		(48.2–49.6)	(5.28–5.73)	(5.28–5.35)			(50.7–52.5)	(3.87–4.42)	(4.03–4.11)	
MetSyn	344	54.9	4.5^g^	4.3	20.0	420	52.5	4.3^h^	4.2	17.6
		(53.5–56.3)	(4.03–4.85)	(4.23–4.34)			(51.3–53.7)	(3.95–4.73)	(4.12–4.24)	
**Elevated triglycerides**										
No MetSyn	121	51.5	5.3^i^	5.1	16.5	323	48.0	3.9^j^	3.8	19.5
		(49.2–53.6)	(4.55–6.09)	(4.96–5.18)			(46.6–49.4)	(3.53–4.35)	(3.76–3.88)	
MetSyn	261	55.5	4.6^k^	4.4	16.9	389	52.6	4.3^l^	4.2	17.7
		(53.9–57.0)	(4.14–5.03)	(4.38–4.50)			(51.4–53.7)	(3.89–4.69)	(4.09–4.21)	
**Low HDL cholesterol**										
No MetSyn	304	41.8	6.3^m^	6.1	10.9	107	41.1	4.7^n^	4.4	11.2
		(40.6–43.1)	(5.68–6.79)	(6.03–6.20)			(38.5–43.8)	(4.00–5.48)	(4.26–4.50)	
MetSyn	238	52.4	4.4^o^	4.2	20.6	180	49.5	4.7^p^	4.5	13.9
		(50.9–54.0)	(3.99–4.88)	(4.13–4.27)			(47.6–51.3)	(4.07–5.27)	(4.37–4.55)	
**Elevated blood pressure**										
No MetSyn	472	57.5	4.7^q^	4.5	20.3	669	51.7	4.0^r^	3.9	20.0
		(56.4–58.6)	(4.23–5.09)	(4.44–4.56)			(50.6–52.8)	(3.69–4.27)	(3.87–3.95)	
MetSyn	245	57.0	4.2^s^	4.1	23.3	368	53.3	4.2^t^	4.1	17.7
		(55.3–58.8)	(3.70–4.68)	(4.02–4.15)			(52.0–54.5)	(3.76–4.63)	(4.00–4.12)	
**Elevated glucose**										
No MetSyn	15	54.3	4.5^u^	4.9	26.7	33	54.0	5.26^v^	5.0	12.1
		(49.1–60.3)	(1.64–7.15)	(4.57–5.31)			(49.5–58.5)	(3.91–6.97)	(4.84–5.23)	
MetSyn	34	63.2	3.1^w^	3.0	41.2	61	56.9	3.5^x^	3.5	24.6
		(58.9–67.3)	(1.91–4.38)	(2.88–3.18)			(54.1–59.4)	(2.67–4.39)	(3.34–3.57)	

Data are presented as mean with 95% Confidence interval (CI). n: number of participants fulfilling the specific criteria; No MetSyn: participants not fulfilling the specific criteriafor metabolic syndrome; MetSyn: participants fulfilling the specific criteria for metabolic syndrome; ED: endothelial dysfunction (FMD≤0%); FMD: flow-mediated dilation, as percent dilation from baseline diameter; FMD_adj_: FMD adjusted for age; HDL: high density lipoprotein. Annotations with letters on different FMD groups to indicate p-values for differences as follows (only significant differences listed): a different from b, c and d, p<0.001; e different from f and g and h, p<0.001; i different from j, p = 0.001; i different from l, p = 0.013; j different from k, p = 0.045; l different from m, p = 0.002; m different from o and p, p<0.001; m different from n, p = 0.002; q different from r, p = 0.007; v different from w, p = 0.028; v different from x, p = 0.040.

**Table 2:** Flow-mediated dilation and endothelial dysfunction by the metabolic syndrome and blood glucose stratified by gender.

	Met Syn	Glucose	n	FMD	(95% CI)	FMD_adjusted_	(95% CI)	ED, %
**Women**			**2528**	**5.33**	**(5.16–5.51)**			**16.0**
	No	Normal	2166	5.48^a^	(5.28–5.67)	5.25	(5.21–5.27)	15.3
	No	Elevated	15	4.47^b^	(1.48–7.46)	4.94	(4.57–5.31)	26.7
	Yes	Normal	313	4.61^c^	(4.18–5.03)	4.46	(4.39–4.51)	17.6
	Yes	Elevated	34	3.11^d^	(1.86–4.30)	3.03	(2.88–3.10)	41.2
**Men**			**2211**	**4.29**	**(4.13–4.45)**			17.3
	No	Normal	1743	4.27^e^	(4.09–4.45)	4.16	(4.13–4.19)	17.5
	No	Elevated	33	5.26^f^	(3.64–6.89)	5.04	(4.84–5.23)	12.1
	Yes	Normal	374	4.42^g^	(4.00–4.83)	4.25	(4.18–4.31)	16.3
	Yes	Elevated	61	3.49^h^	(2.62–4.30)	3.46	(3.34–3.50)	24.6

Met Syn: metabolic syndrome; n: number of participants in each category; FMD: flow-mediated vasodilation; FMD_adjusted_ :FMD adjusted for age; ED: endothelial dysfunction; CI: 95% confidence interval. Annotations as letters on FMD groups to show differences between groups and p-values, with differences as follows with ANOVA: a different from c, d, e, g and h (p<0.005); c different from d (p = 0.066) and trend for h (p = 0.057);d different from b and f (p = 0.036); f trend with h (p = 0.051).

### Blood glucose

**Figure 2:**
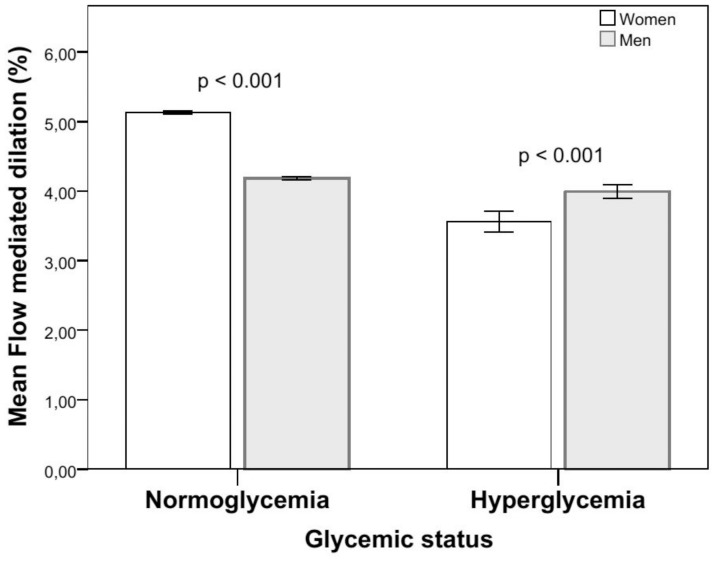
Gender difference in flow-meditated dilation with glycemic status. Mean flow-mediated dilation adjusted for age and its dependence on glycemic status are shown separately for women and men. Vertical lines indicate 95% confidence intervals. Difference between normo-and hyperglycemic women, p = 0.003, men p = 0.91.

Regardless of other risk factors, FMD in women with hyperglycemia was lower (p = 0.005) and prevalence of ED (p = 0.004) higher than in women with normoglycaemia. This was not observed in men (Table 2, Fig. 2; differences in men with hyperglycemia vs normoglycaemia: FMD, p = 0.65 and ED, p = 0.47). Odds ratios for ED in subjects with hyperglycemia were 2.78 (95% CI: 2.57–2.99) in women and 1.20 (1.12–1.28) in men (Table 3). For both genders, the prevalence of ED in hyperglycemic subjects was markedly higher than in subjects with the metabolic syndrome (Table 2, Table 3). In women with the metabolic syndrome, there was a marked difference in FMD (p<0.001) and prevalence of ED (p = 0.004) between those with hyperglycemia and those with normoglycaemia. In hypertensive women, but not in hypertensive men, FMD was reduced in subjects with concomitant hyperglycemia, compared to hypertensive subjects with normoglycaemia (p = 0.02, Table 4).

**Table 3:** Odds ratios for endothelial dysfunction, single predictors^a^.

	Both genders		Women		Men	
	OR	(95% CI)	OR	(95% CI)	OR	(95% CI)
**Met Syn**	1.20	(1.16–1.23)	1.34	(1.29–1.39)	1.07	(1.03–1.11)
**Unfit**	1.31	(1.28–1.34)	1.54	(1.46–1.55)	1.12	(1.09–1.16)
**Smoking**	1.29	(1.26–1.32)	1.44	(1.40–1.49)	1.13	(1.09–1.17)
**High blood pressure**	1.30	(1.28–1.33)	1.33	(1.30–1.36)	1.28	(1.25–1.31)
**Stage 1 hypertension**	1.45	(1.41–1.50)	1.57	(1.51–1.64)	1.31	(1.26–1.36)
**Stage 2 hypertension**	1.82	(1.26–2.08)	2.40	(2.25–2.56)	1.28	(1.20–1.38)
**High blood pressure+smoking** b	1.65	(1.57–1.74)	2.10	(1.95–2.25)	1.35	(1.26–1.45)
**Hyperglycemia**	1.69	(1.61–1.78)	2.78	(2.57–2.99)	1.20	(1.12–1.28)
**Low HDL**	0.88	(0.85–0.90)	0.95	(0.92–0.99)	0.78	(0.74–0.82)
**Low VO_2 peak_**	1.99	(1.92–2.06)	2.69	(2.56–2.83)	1.52	(1.45–1.59)
**Low VO_2 peak_+high blood pressure+smoking** c	2.15	(2.00–2.32)	2.68	(2.41–2.97)	1.76	(1.59–1.95)

All listed predictors are significant for endothelial dysfunction (p<0.05). OR:odds ratio; CI: 95% confidence interval; MetSyn: metabolic syndrome; unfit: VO_2 peak_ adjusted for body weight below median for study population; high blood pressure: systolic blood pressure ≥130 and/or diastolic blood pressure ≥85 mmHg (millimeters mercury); Stage 1 hypertension: Systolic blood pressure ≥140 and <160 mmHg or diastolic blood pressure ≥90 and <100 mm Hg; Stage 2 hypertension: Systolic blood pressure ≥160 and <180 mmHg or diastolic blood pressure ≥100 and <110 mm Hg; low HDL: high-density lipoprotein cholesterol <1.0 mmol/L in men and <1.3 mmol/L in women; low VO_2 max_: lowest gender specific quartile compared to the highest adjusted for body weight.

a interaction variables included in term “single predictor”;

b interaction variable between high blood pressure and smoking;

c interaction variable between low VO_2peak_, high blood pressure and smoking. Values are adjusted for age.

**Table 4:** Flow-mediated dilation and endothelial dysfunction by elevated glucose and blood pressure stratified by gender.

	Elevated glucose	Elevated blood pressure	n	Age	FMD	FMD_adjusted_	ED, %
				(CI)	(CI)	(CI)	(FMD≤0%)
**Women**	No	No	1789	45.1	5.68^a^	5.48	13.7
				(44.4–45.7)	(5.47–5.87)	(5.44–5.51)	
	No	Yes	690	57.1	4.57^b^	4.43	20.4
				(56.1–58.0)	(4.25–4.90)	(4.38–4.47)	
	Yes	No	22	55.4	4.51^c^	4.61	27.3
				(49.7–61.9)	(2.59–6.32)	(4.34–4.87)	
	Yes	Yes	27	64.5	2.73^d^	2.82	44.4
				(60.6–68.2)	(1.36–4.25)	(2.65–2.99)	
**Men**	No	No	1136	46.1	4.49^e^	4.37	15.6
				(45.3–46.9)	(4.26–4.71)	(4.33–4.40)	
	No	Yes	981	51.9	4.08^f^	3.98	19.0
				(51.0–52.8)	(3.82–4.33)	(3.94–4.01)	
	Yes	No	38	51.4	4.66^g^	4.34	15.8
				(48.3–54.6)	(3.41–6.12)	(4.17–4.51)	
	Yes	Yes	56	58.9	3.73^h^	3.79	23.2
				(55.9–62.2)	(2.73–4.67)	(3.66–3.92)	

FMD:flow-mediated dilation; FMD_adjusted_: predicted values of FMD adjusted for age; ED: endothelial dysfunction (FMD≤0%); elevated glucose: blood glucose ≥7.8 mmol/L; elevated blood pressure: systolic blood pressure ≥130 mmHg and/or diastolic blood pressure ≥85 mmHg; n: numbers of participants; CI: 95% confidence interval. Annotations as letters on FMD groups to show differences between groups and p-values, with differences as follows with ANOVA;Difference with p<0.001; a from b, d, e, f and h, with p<0.05; a from c, d from b and e, f from b and e.pone.0101371.g004.tif

### Blood pressure

**Figure 3:**
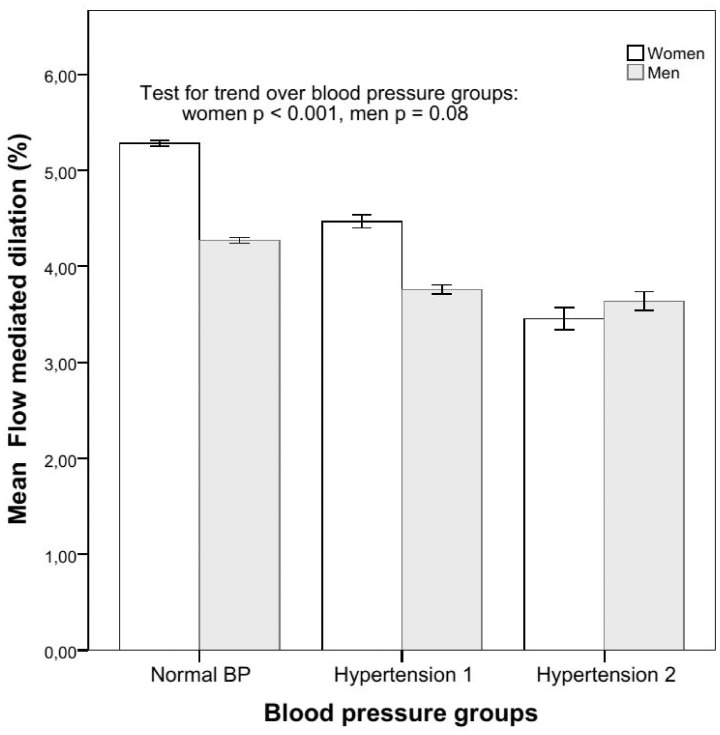
Flow-mediated dilation in normotension and hypertension class I and II. Mean flow-mediated dilation adjusted for age and its dependence with blood pressure groups are shown separately for women and men. Vertical lines indicate 95% confidence intervals.

In women, the correlation between FMD and systolic blood pressure (SBP) was −0.13, and the regression coefficient −0.11 (p<0.001); in men the correlation was −0.07 and the regression coefficient −0.09 (p<0.05); difference: z = −2.06, p = 0.02. Odds ratios for ED in subjects with hypertension were higher in women than in men (Table 3), and markedly so in subjects with stage II hypertension (systolic blood pressure ≥160 and <180 mm Hg or diastolic blood pressure ≥100 and <110 mm Hg) with an odds ratio of 2.40 (95% CI: 2.25–2.56) in women and 1.28 (1.20–1.38) in men. In the range of systolic blood pressures from 100 mmHg to 165 mmHg, FMD declined with increasing blood pressures (p<0.001, Fig. 3) in women, but not in men. In the same range, the prevalence of ED was also higher with increasing blood pressure (p<0.001) in women, but not in men (Table 1).

### Other risk factors

The correlation between fitness (VO_2 peak_) and FMD was 0.124 (p<0.001) in women and 0.053 (p = 0.016) in men (difference: z = 2.44, p = 0.007). Odds ratios for ED in subjects with low fitness were 1.54 (95% CI: 1.46–1.55) in women, and 1.12 (1.09–1.16) in men (Table 3).

Odds ratio for ED was higher in female smokers than in male smokers (1.44, 95% CI: 1.40–1.49 vs. 1.13, 95% CI: 1.09–1.17). When adjusting regression models for systolic blood pressure and VO_2 peak_, smoking was no longer associated with low FMD in men, but persisted in women. More detailed analyses of the effect on endothelial function by smoking habits will be reported in a separate study.

Overweight and obesity had no impact on FMD or the prevalence of ED in either gender. Significant predictors for ED are listed in Table 3.

## Discussion

The main finding of the current study was that hyperglycemia, high blood pressure, low fitness and a cluster of cardiovascular risk factors comprising the metabolic syndrome are more strongly associated with reduced flow-mediated vasodilation (FMD) and endothelial dysfunction (ED) in women than in men.

### Metabolic factors

It has previously been shown that mortality from CVD is higher in subjects with the metabolic syndrome [23], and that this association is stronger in women than in men [13], [23]. It has also been suggested that hyperglycemia per se, more than the metabolic syndrome as an entity, predicts the prognosis and outcome in CVD [7], [18], [24], [25], [26], and that young women are especially prone to the negative effects of both [27]. The present study shows that FMD was lower and prevalence of ED was higher in women with hyperglycemia. This was not the case in male counterparts. In women with the metabolic syndrome, there was also a marked difference in FMD and the prevalence of ED between those with hyperglycemia and those with normoglycaemia. Estrogens have been suggested as a key mechanism of women’s lower relative cardiovascular disease risk; possibly by mobilizing endothelial progenitor cells from the bone marrow and protection from apoptosis [28]; insulin resistance has been negatively correlated with the number of endothelial progenitor cells [29]. Increased formation of advanced glycation end products (AGE) is regarded as one of the main mechanisms of vascular damage in patients with diabetes [30]. These products affect cell function by binding to their receptor [31], thereby inducing endothelial dysfunction. Women usually have lower incidence of heart disease prior to menopause, suggesting that estrogen may protect towards endothelial dysfunction and heart disease. Some studies have shown a gender difference, with healthy young women presenting higher plasma levels of AGE compared to their male counterparts [32], [33]. Another study has shown that estrogens inhibit the synthesis of AGE in vaginal epithelial tissues of postmenopausal women [34]. Thus, the role of estrogen regulating AGE signaling with regard to gender differences remains unclear [35].

It may also be that women have to undergo a greater metabolic deterioration than men before insulin resistance, impaired regulation of blood glucose [30], and endothelial dysfunction ensue. Alternatively there may be a gender difference in endothelial expression of specific genes when exposed to hyperglycemia [31]. Our findings support the notion that endothelial dysfunction is an important mechanism of gender-specific effects on CVD in patients with the metabolic syndrome, hyperglycemia or diabetes mellitus. Furthermore, the metabolic syndrome used as an entity may mask important differences in the sensitivity to specific risk factors, especially hyperglycemia, in assessing health and mortality [24], [25], [32].

We observed a trend for association between reduced FMD and increasing number of metabolic syndrome risk factors also in men, but the association did not reach the level of significance (Figure 1). This finding may be due to exclusion of participants with more than three risk factors because of clinically manifest CVD or drug treatment of hypertension, thereby underestimating the association between the metabolic syndrome and endothelial dysfunction. However, this may also apply to women with five vs. four risk factors, underestimating the association in women as well.

### Hypertension

The present study is the first to demonstrate a linear decrease in FMD with increasing systolic blood pressure in self-reported healthy women and with stronger association in women than in men. The prevalence of ED was higher with increasing blood pressure only in women. The strong association between hypertension and CVD mortality and morbidity is well known [33], [34], [35], but there is a lack of knowledge regarding gender-specific effects of hypertension. Our results suggest that hypertension is an important determinant of endothelial function in both genders, and that it may have a larger impact in women. Hypertension is strongly associated with increased mortality from ischemic heart disease in patients with type 2 diabetes [36], [37], especially in women [38]. As illustrated in Table 4, our findings suggest that hyperglycemia negatively affects endothelial function in hypertensive subjects.

### Fitness and obesity

The correlation between FMD and VO_2peak_ was small, though significant, and larger in women than in men; the age-adjusted odds ratio for fitness was three times higher with FMD in the lowest quartile compared to the highest in women but not in men. Thus, low fitness may affect endothelial function more negatively in women than in men. These findings concur with previously reported protective associations of exercise with CVD and mortality [23], [39], [40]; and with recent studies demonstrating larger effects in women than in men [39], [41]. In contrast, we found that overweight and obesity had no impact on FMD or the prevalence of ED in either gender. In women, obesity had a negative effect on FMD only in the presence of the metabolic syndrome. Therefore, obesity is not included in Table 3 as a significant predictor. Although we only measured body weight and waist circumference, these results suggest that isolated overweight and obesity are not likely to be independent risk factors for CVD via their effects on endothelial function. This is in contrast with previous findings suggesting an association between these factors and increased CVD mortality and morbidity [39], [42], especially in middle-aged women [43], [44], [45], [46].

### Limitations

The Fitness Study was part of an ambulatory health survey with a large number of participants who came for examinations at all times of the day, some directly from work. Although pre-circulated information asked them to be fasting, they were allowed a snack while waiting. As a result, 75% reported food intake less than 4 hours before testing. Lacking a standard cut-off for this situation, we used the criterion for impaired glucose tolerance of ≥7.8 mmol/L two hours after an oral glucose tolerance test for all participants. This cut-off is conservative and may have underestimated the number with the metabolic syndrome. The questionnaire data on oral contraceptives and hormone replacement was self-reported and not sufficiently detailed to be used for statistical adjustments.

## Conclusion

In self-reported healthy subjects, the reduction in endothelial function in women was more strongly associated with hyperglycemia, hypertension, low fitness and the metabolic syndrome than in men. This gender difference in endothelial function may explain why the metabolic syndrome, and especially hyperglycemia, is associated with higher cardiovascular risk and worse prognosis in women.
